# How Can Hypnodontics Manage Severe Gag Reflex for Root Canal Therapy? A Case Report

**DOI:** 10.7508/iej.2016.02.015

**Published:** 2016-03-20

**Authors:** Mohsen Ramazani, Nafiseh zarenejad, Masoud Parirokh, Samir Zahedpasha

**Affiliations:** a *Department of Endodontics, Dental School, Mazandaran University of Medical Sciences, Sari, Iran, and Iranian Scientific Society of Clinical Hypnosis, Tehran, Iran; *; b * Department of Restorative Dentistry, Dental School, Mazandaran University of Medical Sciences, Sari, Iran; *; c * Oral and Dental Research Center, Dental School, Kerman University of Medical Sciences, Kerman, Iran; *; d * Department of Oral and Maxillofacial Medicine and Diagnostic Sciences, Case Western Reserve University, School of Dental Medicine, Cleveland, OH, USA*

**Keywords:** Dental Treatment, Endodontics, Gag Reflex, Hypnosis, Root Canal Therapy

## Abstract

In endodontics, severe involuntary gagging can have a severe impact on treatment procedure. There are many ways to ease the gag reflex, one of which is hypnosis. A 34-year-old male was referred for root canal treatment of a molar tooth. He had not received any dental treatments for the past nine years due to fear of severe gag reflex. Three hypnotic sessions based upon eye fixation, progressive muscle relaxation and guided imagery techniques were spent for psychosomatic management. The gag reflex was controlled and reduced to a normal level, and the required dental treatments including root canal therapy and restoration were performed successfully. This report shows that hypnosis can control gag reflex for dental treatments.

## Introduction

Gag reflex is considered as protective reaction originating from the cortical level to preclude the entry of any foreign body into the upper respiratory tract [1]. It may also be an acquired reflex triggered by various stimuli such as visual, olfactory, acoustic, psychic, chemical, or even toxins transmitted through the blood circulation. Briefly it can be elicited by the dentist’s fingers or instruments contacting the oral mucosa or even by non-tactile stimuli, for instances, visiting a dentist or remembering a previous dental experience [2].

A pronounced gag reflex compromises all aspects of endodontic treatment from diagnosis, clinical examination, radiography, to the treatment interventions [2, 3]. It makes the tolerance of any instrument such as injection syringe, ejector, dental dam or x-ray sensor/film difficult, if not impossible and so the acceptable and comfortable endodontic treatment may not be provided [3]. As the worst scenario, marked gagging reflex, may lead to avoidance of treatment or dental care forever [4]. Distraction techniques such as conversation with the patient, concentration on breathing and raising the foot, may be used to control the mild to moderate gagging [1, 2, 5]. Other methods include the use of topical anesthetic sprays or salt, modifying the instruments needed such as impression trays, systematic desensitization and acupuncture [5]. 

Severe gagging in dentistry may also be managed by pharmacological methods including intravenous sedation, glossopharyngeal nerve block or non-pharmacological modalities such as cognitive behavior therapy (CBT), cognitive behavior hypnosis (CBH), or hypnosis [3, 6]. Previous case presentations and reviews mostly reported using hypnosis for management of gag reflex in patients who had intolerance to dentures and various dental practices [7]. In endodontic practice, hypnosis was reported to be effective in reducing anxiety [8]. The present case shows the beneficial utilization of hypnosis to manage severe gag reflex for performing root canal treatment.

## Case Report

A 34-year-old male referred to our clinic suffering from two simultaneous problems; dull pain and swelling originated from mandibular first molar and severe gag reflex. Common oral examination was difficult due to his marked gagging. Using instant hypnotic inductions, tooth sensibility tests were implemented which showed negative response of the target tooth to heat, cold and electric pulp tester (Analytic Technology, Redmond, WA, USA). An intraoral vestibular swelling adjacent to the tooth was visible. Percussion test led to a positive response. In the extra-oral radiography of the involved tooth, a periapical lesion around the involved tooth could be observed. Pulp necrosis with acute apical abscess was the diagnosis. Endodontic treatment was suggested to the patient. 

This patient had marked gag reflex in dental office for the past nine years. Using *Hypnotalk* it was unfolded that visiting a dentist or remembering the previous dentistry experiences elicits severe gagging at the time dissuading from routine dental care or even emergency dental services repeatedly. Hypnosis was suggested to the patient to provide an effective atmosphere for conduction of root canal treatment.

Assessment of gagging was done before hypnotic intervention based on gagging severity scale presented by Dickinson and Fiske [2]: 

*grade I* (normal gagging reflex), some dental interventions such as taking the upper jaw impression or restoring the lingual, palatal, or distal sides of upper molars can elicit gagging which is considered as normal, and is controlled by the patient himself; 

*grade II* (mild gagging), impression taking, filling, scaling, or taking intra-oral radiography may occasionally lead to mild gagging, with or without assistance or reassurance, the patient can continue the treatment procedure; 

*grade III* (moderate gagging), gagging resulted from routine dental interventions such as examination of susceptible parts of oral cavity as lower molars would follow cessation of the measures, otherwise treatment planning might be negatively influenced, and treatment would be stopped; 

*grade IV* (severe gagging), even a simple visual evaluation can render sever gagging. Without special supportive measures, treatments options may be missed and treatment planning would not be completed;

*grade V* (very severe gagging), patient’s attendance or behavior such as remembering the previous dental experiences are among predisposing factors, meaning that no physical intervention is needed to elicit gagging. 

Psychological or behavior management may be used to control the confounding gagging, otherwise the treatment options are really limited, and treatment planning is adversely influenced. According to this classification, our patient was categorized as *grade IV*. No contributory medical or psychological history was observed.

Preliminarily, three sessions of hypnosis were preplanned to cover the problem. Hypnosis interventions aimed at stress management, memory re-patterning through substitution of negative previous dental experiences with positive, virtual visualization about dental treatment without disturbing gag reflex, *sterognosis* trial (haptic perception is defined as capability of perceiving and recognizing an object form without visual and auditory help, through using tactile information about spatial specification, temperature, size and texture), reinforcement of self-confidence and reminding the patient of his critical role in dental communication. Due to patient’s excellent collaboration, at the end of the third session, necessary treatments including root canal therapy and permanent amalgam restoration of the given tooth were completed successfully. Treatment options informed consent the steps respectively done are as follow:

***First session:*** *Pre talk* or *Hypnotalk* is the most important factor to build an effective hypnotic rapport. It includes evaluation of mental and psychological status, discovery of the elements affecting the experience interpretation and acceptation and reassuring the patient that previously-existent negative memories can be sensed positively through hypnosis and mental remodeling at present and in the future. After such conversational exploration, a 50-min hypnotic trance with a light background music was done to donate a pleasant and stress-free experience on dental chair to the patient, and separate him from his previous dental disturbing experiences especially severe gagging, with help of *sterognosis*. The latter was secured with placing some dental instruments and materials relevant to endodontics, in the oral cavity and the patient was asked to feel and identify those with the tongue.

In fact, it was conducted through building a virtual dental atmosphere mainly focusing at gagging-free dental treatment. At the end of the first trance the patient was conditioned to have a deeper and more pleasant hypnosis at the next session which was set one day later. Additionally the patient was trained to have progressive muscle relaxation (PMR) before night sleeping, later on.

For pain control, a nonsteroidal anti-inflammatory drug (NSAID) was suggested to be used on demand especially 1 hour prior to the second session in order to decrease the postoperative pain. 

***Second session*** **:** Just one day after the first visit, using the conditional key, a deeper and faster sound trance was achieved upon which a brief virtual experience of gag-free dental treatment presented to the patient immediately. Then, again a real desensitization technique was utilized, meaning that through hypnotic trance, some dental instruments and devices such as injection syringe, mirror, explorer, radiography digital sensor or dental film and isolation equipment, were moved to lightly contact the high-risk tissues including lips, anterior and then posterior parts of oral cavity, so that the patient himself can accept the possibility of having such an experience. At the end of the second session when the patient had deeply been fallen into hypnosis, the IANB anesthesia using lidocaine 2% and epinephrine 1:80000 (Persocaine-E, DaruPakhsh Pharmaceutical Co., Tehran, Iran) was administered, and when appropriate anesthesia was achieved, the tooth was isolated by placing a rubber dam and the root canals were cleaned and shaped by Mtwo rotary instruments (VDW, Munchen, Germany). Irrigation was done using 2.5% sodium hypochlorite (Golrang, Tehran, Iran) and the tooth was temporarily restored using Cavisol (Golchai, Tehran, Iran) following intra-canal dressing with calcium hydroxide (Kimia, Tehran, Iran).

The next session was scheduled for one week later. For homework, the patient was asked to do the relaxation exercises and *sterognosis* with common objects, such as a toothbrush, spoon, fork, *etc.* to strengthen the new intra-oral sensibility establishment. The patient was given a mild analgesic to be used on demand.

***Third session:*** After a week, the patient attended the office reporting no pain between the visits. Each night relaxation homework had helped him to have a pleasant and enjoyable sleep. It was surprisingly revealed that the gag threshold has been reversed to *grade I* of Dickinson and Fiske classification. In deep hypnotic trance and without gagging severely, after irrigation with 2.5% sodium hypochlorite, the canals were dried and obturated. Finally, the crown was permanently restored with amalgam.

Since treatment completion, the patient is on a regular recall program. He is now able to control his swallowing and gag reflex well. No adverse side effect was reported after three sessions of hypnosis coverage. All the treatment procedures had been recorded after taking permission from the patient to be shown on scientific purposes.

## Discussion

Patients with exaggerated gag reflex may experience various problems during dental treatments. Patient would be unable to tolerate the dental dam, x-ray sensor/film, saliva ejector, or even injection syringe or small dental instruments. The present patient had such a situation which was successfully managed through using hypnosis prior and during root canal treatment [1]. 

Sedation and hypnosis are available modalities for controlling sever gagging, owing their own advantages and disadvantages. For instance, sedation cannot be administered for not all patients, whereas the hypnosis for management of severe gag reflex can be used just for highly hypnotizable cases. Fortunately, this case was cooperative due to being highly hypnotizable. Sedation is not as time consuming as hypnosis, although it is technique sensitive. On the other hand, sedation is not cost effective, since it needs special equipment, contrary to hypnosis. The most prominent priority of hypnosis to sedation is the depth of its effectiveness and consequently durability, meaning that hypnosis is able to help patients with severe gag reflex overcome their gagging for a longer time or even for the rest of life and so the patient regularly can attend dental office, while with using sedation, patient would not get rid of the marked gagging which temporarily bypassed and remains as a contributing problem. Sedation has some complications and needs special care while hypnosis is safe.

In addition, another issue to opt between sedation and hypnosis is patient’s preference. Our patient selected hypnosis and if not effective, sedation would have been the next choice [9, 10].

There are instant hypnosis inductions which take a shorter period of time, but have less comprehensive and durable effectiveness compared with classic inductions. In the present case, both modalities were utilized depending on the benefits anticipated. Moreover, the subject’s hypnotizability must be considered. Although for complicated cases such as severe gagging high hypnotizability is needed, the patients with medium or even low hypnotizability can also benefit from hypnosis for stress and anxiety management, hypnoanalgesia or hypnoaneshtesia [11, 12]. Even those without previous hypnosis experiences can be hypnotized for clinical purposes [13, 14]. Our case was from the first group with high susceptibility for hypnosis intervention. 

In the present case, treatment was completed in three consecutive visits. The first visit focused on preparing the patient for hypnosis in which a suitable dental situation is to be provided, and the following two visits to perform root canal treatment and restoration. The treatment could be limited to two visits if the patient had no apical abscess that should be managed by root canal cleaning and shaping in order to provide a condition to complete root canal therapy. 

Eventually it is necessary to mention that according to the official hypnosis association policies, the dentists who are interested in hypnosis must have enrolled, participated into, and gain official certificate from the hypnosis associations worldwide.

## Conclusion

Gag reflex can be complicate dental treatment prevent optimal treatment results. Based on psychology and psychiatry researchthe psychotic part is closely integrated with the somatic part of medical conditions including gag reflex. This report shows that in case of severe gag reflex, especially with pronounced psychological background, hypnosis can be a safe, effective, and user-friendly method to modify patient's sensation, memory, and compliance.
